# Decision-Making by Patients With Methamphetamine Use Disorder Receiving Contingency Management Treatment: Magnitude and Frequency Effects

**DOI:** 10.3389/fpsyt.2020.00022

**Published:** 2020-02-18

**Authors:** Marilyn T. Lake, Steven Shoptaw, Jonathan C. Ipser, Sae Takada, Lara J. van Nunen, Gosia Lipinska, Dan J. Stein, Edythe D. London

**Affiliations:** ^1^ Department of Psychiatry and Mental Health, University of Cape Town, Cape Town, South Africa; ^2^ Department of Psychology, University of Cape Town, Cape Town, South Africa; ^3^ Department of Family Medicine, University of California, Los Angeles, Los Angeles, CA, United States; ^4^ Division of General Internal Medicine and Health Services Research, Department of Medicine, University of California, Los Angeles, Los Angeles, CA, United States; ^5^ Veterans Health Services Research and Development Service (VA HSR&D) Center for Study of Healthcare Innovation, Implementation, & Policy, Los Angeles, CA, United States; ^6^ SA MRC Unit on Risk & Resilience in Mental Disorders, Department of Psychiatry and Neuroscience Institute, University of Cape Town, Cape Town, South Africa; ^7^ Department of Psychiatry and Biobehavioral Sciences, Department of Molecular and Medical Pharmacology, and the Brain Research Institute, University of California, Los Angeles, Los Angeles, CA, United States

**Keywords:** decision-making, risk-taking, methamphetamine, methamphetamine use disorder, Iowa Gambling Task, contingency management

## Abstract

**Background:**

Individuals with substance use disorders exhibit maladaptive decision-making on the Iowa Gambling Task (IGT), which involves selecting from card decks differing in the magnitudes of rewards, and the frequency and magnitude of losses. We investigated whether baseline IGT performance could predict responses to contingency management (CM) by treatment-seeking individuals with methamphetamine use disorder (MA Use Disorder) in Cape Town, South Africa.

**Methods:**

Twenty-nine individuals with MA Use Disorder underwent an 8-week, escalating reinforcement, voucher-based CM treatment in a study on the suitability of CM therapy for the South African context. Along with 20 healthy control participants, they performed a computerized version of the IGT before starting CM treatment. Seventeen participants maintained abstinence from methamphetamine throughout the trial (full responders), and 12 had an incomplete response (partial responders). Performance on the IGT was scored for magnitude effect (selection of large immediate rewards with high long-term loss) and for frequency effect (preference for frequent rewards and avoidance of frequent losses). Group differences were investigated using linear mixed-effect modeling.

**Results:**

Partial responders made more selections from decks providing large, immediate rewards and long-term losses than healthy controls [*p* = 0.038, *g* = -0.77 (-1.09: -0.44)]. Full responders showed a greater, nonsignificant preference for frequent rewards and aversion to frequent losses than partial responders [*p* = 0.054, *g* = -0.63 (-0.95: -0.29)].

**Conclusions:**

A predilection for choices based on the size and immediacy of reward may reflect a cognitive strategy that works against CM. Pretesting with a decision-making task, such as the IGT, may help in matching cognitive therapies to clients with MA Use Disorder.

## Introduction

Substance misuse is linked to maladaptive risk taking that typically results in long-term loss or foregone gain in the context of uncertainty (1, 2). Such decision-making deficits have been observed in individuals with substance use disorders using the Iowa Gambling Task (IGT), a laboratory test of adaptive decision-making (3–6), and on temporal discounting tasks, which evaluate a participant’s devaluation of rewards as a function of delay (7, 8).

On the IGT, individuals who have or are at risk for drug use disorders demonstrate maladaptive decision-making by differentially favoring choices associated with large, immediate rewards over choices that produce long-term gain, as compared with individuals who do not use drugs of abuse or who are not at risk (3, 6, 9). This finding is consistent within methamphetamine-dependent samples [Diagnostic and Statistical Manual of Mental Disorders, Fourth Edition (DSM-IV)] in particular Gonzalez et al. (5) and van der Plas et al. (2). The IGT has been used predominately to assess the influence of the *immediacy* and *magnitude* of rewards (and losses) on decision-making, but it has also been used to investigate the impact of the *frequency* with which rewards and losses are presented (10, 11). Chiu et al. (12) found that healthy individuals demonstrate a tendency to seek out gains that occur more *frequently* rather than exclusively seeking out long-term gains, as initially proposed by Bechara et al. (13). On a different task, Voon et al. (14) found that methamphetamine-dependent individuals (DSM-IV) made riskier choices for lower probability/frequency rewards and higher probability losses than healthy controls, suggesting that decision-making of methamphetamine use disorder (MA Use Disorder) patients may also be influenced by outcome frequency. However, this has yet to be investigated with respect to the IGT.

Decision-making deficits appear to vary depending on the substance in question. Ahn et al. (15) found that amphetamine-dependent (DSM-IV) individuals exhibit maladaptive decision-making that is characterized by a greater sensitivity to reward, whereas heroin-dependent individuals demonstrate less sensitivity to loss as compared with healthy individuals. Therefore, it is necessary to assess decision-making deficits by substance type. Decision-making deficits have also been shown to vary within same substance-using samples, with associated treatment implications (16, 17). Chen et al. (18) found that poorer performance on the IGT by methamphetamine-dependent (DSM-IV) individuals predicted dropout in cognitive behavioral therapy (CBT), while better performance on the IGT was related to greater treatment retention. Evidence of variability in decision-making deficits among individuals with substance use disorders and its links to treatment outcomes suggests the need for greater assessment of a potential spectrum of decision-making profiles *within* substance use disorder populations, but this relationship may also be specific to the type of treatment, and treatment outcome in question.

There is limited research into the relationship between maladaptive decision-making in MA Use Disorder and risk of relapse in the context of contingency management (CM). A behavioral treatment that rewards abstinence with rewards, often monetary, CM has greater short-term therapeutic efficacy than other treatments for MA Use Disorder, such as CBT (19). Maladaptive decision-making is particularly relevant to the context of CM treatment, which directly positions the reinforcing value of monetary rewards against the reinforcing value of drugs, measuring an individual’s choices between monetary rewards for abstinence versus continued drug use, which lead to missed monetary rewards (20). The IGT was used at treatment entry to evaluate whether response to CM treatment corresponds with deficits in decision-making as measured by the task.

This project is a key part of a pilot study to evaluate mechanisms of CM therapy for MA Use Disorder patients in Cape Town, South Africa. The objective of this trial is to examine links between maladaptive decision-making using the IGT with CM treatment outcomes and to compare IGT responses for the MA Use Disorder patients with a comparable sample of healthy controls. We hypothesized that participants with MA Use Disorder who failed to respond completely to 8 weeks of CM (partial response) would demonstrate significant maladaptive decision-making at baseline relative to participants who responded completely (full response) or to healthy controls as measured by a “magnitude effect” (preference for riskier choices with larger, immediate rewards and long-term losses than for less risky choices). We also predicted that compared to participants with complete CM response and healthy controls, participants who showed partial response to CM would demonstrate greater preference for frequent rewards and avoidance of frequent losses, that is, a “frequency effect.”

## Materials and Methods

### Study Design and Participants

This study was part of a pilot project investigating the suitability of CM in treating MA Use Disorder in South Africa. It used a between-groups, cross-sectional design comparing outcomes to CM (complete response, partial response) among 29 individuals diagnosed with MA Use Disorder (DSM-5) to 20 healthy control participants, see Okafor et al. (21). All participants completed neurocognitive and self-report measures, after which MA Use Disorder patients commenced an 8-week, escalating schedule, thrice-weekly, voucher-based CM intervention. The study protocol was approved by the Health Sciences Human Research Ethics committee of the University of Cape Town and UCLA in accordance with the Declaration of Helsinki. MA Use Disorder patients were recruited through local drug clinics (*n* = 20), and a combination of local newspaper advertisements and snowball sampling was used to recruit additional individuals with MA Use Disorder (*n* = 9) and all healthy control candidates (*n* = 20). Interested candidates provided informed, written consent and were screened for eligibility.

Recruits with suspected MA Use Disorder underwent a 2-week baseline screening period to determine whether they met DSM-5 criteria for MA Use Disorder [Structured Clinical Interview for DSM-5 (SCID) verified by a trained professional], to demonstrate ability to attend thrice-weekly scheduled appointments to provide scheduled urine tests and to confirm recent methamphetamine use, where participants were not made aware of the latter eligibility criterion. Of 269 recruits who were initially screened, 148 individuals were not eligible based on either one of the exclusion criteria outlined under *Screening Tools and Inclusion/Exclusion Criteria*, and a further 88 were excluded from the study due to nonattendance, which was the most common reason for exclusion. From the remaining 33 MA Use Disorder patients who were initially enrolled in CM treatment, four participants were additionally excluded from the CM trial for the following reasons: cocaine use not previously disclosed (*n* = 1), meningitis not previously disclosed (*n* = 1), brain structural abnormality (*n* = 1), and a MA-positive (methamphetamine-positive) urine test at the time of task assessment (*n* = 1). A total of 29 adult MA Use Disorder patients, 18–45 years of age, were enrolled (20 males) in the study.

Participants with MA Use Disorder were categorized according to their response to CM treatment as partial responders (*n =* 12) and full responders (*n =* 17). Full responders were defined as those participants who exclusively presented with MA-negative (methamphetamine-negative) urine samples during CM treatment, demonstrating that they maintained abstinence. Partial responders were defined as those participants who presented with at least one MA-positive or missed urine sample over the entire duration of CM treatment. In addition to verifying methamphetamine use before initiating treatment, urine tests were used to verify treatment response, as well as to verify abstinence from methamphetamine on the day of task assessment, as well as several other substances, including barbiturates, cocaine, opiates, and cannabis, in order to prevent any confounding acute effects of drugs on task performance. If on the day of task assessment a participant presented with a positive urine test for any of the tested substances, the assessment was rescheduled. Participants were abstinent on average 4.2 days before testing on the first day of treatment. Over the CM intervention, partial responders presented with an average of 13.17 negative (i.e., clean) urine samples out of a total of 24 (sd = 6.35), where the remaining 45% represented positive (including missed) urine samples, and 22% of total samples represented missed urine samples. A frequency-matched control group of 20 (13 male) participants who did not use substances of abuse, other than tobacco or occasional alcohol, was enrolled. Matching characteristics included age, education, gender, ethnicity, and broad intellectual function.

### Screening Tools and Inclusion/Exclusion Criteria

All MA Use Disorder patients met diagnostic criteria for current and primary MA Use Disorder, as indicated by the research version of the SCID; secondary use, but not misuse, of cannabis and/or methaqualone (Mandrax) was accepted due to high prevalence of paired use of these substances with methamphetamine in Western Cape, South Africa (22). Tobacco and alcohol use were accepted for both MA Use Disorder and control groups, given typical use of such legal substances within low-socioeconomic communities (23). For methamphetamine-using and control groups, the presence of the following psychiatric comorbidities was exclusionary: non-primary MA Use Disorder or current/past primary substance use disorder involving a substance other than methamphetamine or tobacco, schizophrenia spectrum disorders, bipolar and related disorders, depressive and anxiety disorders not induced by MA Use Disorder, and obsessive–compulsive-related disorders. In addition, primary MA Use Disorder was an exclusion criterion for the control group. For both MA Use Disorder and control groups, antisocial personality disorder was accepted due to its high prevalence in low-socioeconomic status communities in South Africa (24). In addition, performance and verbal subscale scores of the Weschler Abbreviated Scale of Intelligence (WASI) were used to assess capacity to perform neurocognitive tasks. A score of 55 was the minimum requirement to establish competency in understanding task instructions according to local cultural standards (25). Additional exclusion criteria for MA Use Disorder and control groups included use of psychoactive medication that may have potential effects on the central nervous system, current or previous head injury or neurological illness, HIV-seropositive status using a pin-prick test, left-handedness, and limited comprehension of English. MA Use Disorder patients who were unavailable over a 10-week period or required inpatient treatment were not enrolled. As part of the requirements for the neuroimaging component in the broader study, other exclusion criteria for MA Use Disorder patients included current pregnancy, claustrophobia, pacemaker, and metal prosthesis or metal present in the body.

### Contingency Management Intervention Setting

MA Use Disorder patients underwent CM treatment, which required thrice-weekly scheduled clinic visits to provide urine samples, which were analyzed using radioimmunoassay strips (CLIAwaived Inc., San Diego, California, United States) to detect methamphetamine in urine over the prior 48–72 h. Integrity of urine test results was ensured by using supervised urine sample collection, which was further verified using temperature-sensitive strips on collection cups. Participants who provided MA-negative urine samples immediately received vouchers to be redeemed at a large supermarket (Pick n Pay). The value incrementally increased with each subsequent MA-negative urine test, demonstrating continued abstinence to a maximum of 4,850 Rand (USD $404) over the 8 weeks. If a MA-positive urine test was obtained, or if an appointment was missed with no attempt to reschedule the appointment to a future date that was within the number of days it takes to fully metabolize d-amphetamine, participants did not receive a voucher. The next MA-negative urine test following a positive was worth the starting 25 Rand. To sustain motivation, we used a “rapid reset” procedure to return participants to their prior position on the CM schedule following three consecutive scheduled MA-negative urine tests.

### Iowa Gambling Task (IGT)

The Psychology Experiment Building Language (PEBL) 0.14 computerized version of the IGT was used (13). It consists of four virtual decks of cards, A, B, C, and D, each associated with a unique combination of short-term fixed rewards and probabilistic losses, in addition to an associated long-term net payout. Riskier decks (A and B) present short-term high-reward and high-loss contingencies, with consistent choices of such decks yielding low cumulative totals. In contrast, optimal decks (C and D) are linked to short-term low-reward and low-loss contingencies, yielding moderately high cumulative totals. The objective of the IGT is to maximize long-term cumulative payout earned on the task by learning to shift or avoid selection of riskier, disadvantageous decks A and B and favor safer, more advantageous decks C and D within 100 trials.

#### IGT Setting

For both MA Use Disorder and control groups, participants’ vision was first tested using the Snellen chart before administering the IGT. The IGT was administered to participants *via* a desktop computer situated in a quiet, distraction-free room. Participants were instructed to select from four possible virtual decks on screen using a computer mouse, over a total of 100 trials, with the aim of maximizing net gains from the task. Participants were not time restricted, but took approximately 30–45 min to complete the task. Participants were provided with headphones during administration of the IGT in order to hear the sound effects associated with obtaining either a net gain or a net loss on each deck selection.

#### IGT Scoring: Magnitude Effect

The magnitude effect is represented by a greater selection of riskier decks A and B relative to decks C and D. This is indicative of both a greater preference for short-term rewards and an ability to withstand or otherwise lack the foresight of long-term associated losses. It is calculated by summing deck selections from disadvantageous decks and subtracting them from the sum of advantageous deck selections (C + D) – (A + B), with negative scores reflecting the magnitude effect. This net score was calculated for each of four blocks of 20 trials, excluding block 1 (26).

#### IGT Scoring: Frequency Effect

The frequency effect is defined as a greater selection of decks B and D, relative to decks A and C, and demonstrates a preference for frequent short-term rewards and infrequent losses over infrequent rewards and frequent losses (**Table 1**). The frequency effect is calculated as the sum of selections from decks with frequent rewards and infrequent losses minus decks with infrequent rewards and frequent losses (B + D) – (A + C), where higher scores demonstrate the frequency effect. As above, frequency effect scores were generated per block from blocks 2 to 5. Although a preference for frequent rewards and infrequent losses is arguably more optimal than that of infrequent rewards and frequent losses, the frequency effect does not explicitly account for long-term associated outcomes, like the magnitude effect, which is crucial to effective decision-making on the IGT. In turn, the frequency effect may act as a less optimal decision-making strategy, where frequent rewards are sought out and frequent losses avoided without consideration of long-term consequences.

**Table 1:** IGT deck outcome specifications.

	Deck A	Deck B	Deck C	Deck D
Reward magnitude	100	100	50	50
Loss magnitude	150- 350	1250	25-75	250
Long-term average	−250	−250	250	250
Absolute gain-loss frequency	10 gains 5 losses	10 gains 1 loss	10 gain 5 losses	10 gains 1 loss
Net gain-loss frequency	9 gains 5 losses	9 gains 1 loss	9 gains 5 draws	9 gains 1 loss

In order to incentivize performance, a voucher with a flat rate value of 25 Rand, equivalent to USD $2, was offered to participants from both MA Use Disorder and control groups if an overall positive net payoff on the IGT was achieved.

### Linear Mixed-Effect (LME) Modeling of Decision-Making

#### Magnitude and Frequency Effect

Utilizing the nlme package (27) on the R programming platform (28), an LME model was used to test for differences between partial responders, full responders, and controls in both the magnitude effect and frequency effect across blocks 2–5 on the IGT at baseline. LME modeling is advantageous as it allows for the estimation of fixed effects while simultaneously accounting for the clustered or hierarchical structure of data, namely, within-cluster relationships (the random effect). In this study, group differences (the fixed effect) were estimated in conjunction with within-subject variability (random effects), represented by repeated block scores per subject. The model assessed net block score as the unit of observation at level-1, accounting for its clustering within each participant, where the individual participant is specified as the unit at level-2. An LME framework is particularly appropriate given previous findings of within-group heterogeneity in IGT performance of substance-using and healthy samples (1, 10). Each LME model was compared to a fixed-effect model (in absence of random effects) in order to confirm the presence of individual variability.

### *Post Hoc* Contrasts of Group Differences

In order to minimize familywise error associated with multiple group comparisons, *post hoc* contrasts were conducted on all LME models to compare the groups using Tukey’s p-adjustment correction method, carried out with the lsmeans r package (29). Hedges g effect size estimates were estimated using the compute.es r package (30), where hedges g estimation was presented due to its utility in generating unbiased estimates particularly within smaller samples. All hedges g confidence intervals were bootstrapped. Small, medium, and large effect sizes were represented by *g* values of 0.2, 0.5, and 0.8, respectively (31). Findings were considered significant with p-values < 0.05.

### Covariates of Decision-Making

Several sociodemographic, individual, and drug use variables, including sex, education, broad intellectual function, and drug use history have been previously linked with IGT performance (32–38). In order to control for their potential effects on task performance, all covariates were entered as fixed effects into models of magnitude effect and frequency effect, but were only retained if the model was significantly improved with the inclusion of covariates, based on the likelihood ratio.

## Results

### Sample Characteristics

The majority of MA Use Disorder patients and matched controls were self-reported as “colored” (27 and 19, respectively), where the term “colored” loosely describes an ethnic group of persons of mixed European and African or Asian descent, who makes up a substantial proportion of the Western Cape population, where the study took place. A minority of both MA Use Disorder patients and healthy controls were self-reported as “black” (2 and 1, respectively). The three groups (partial responders, full responders, controls) did not differ in sex, age, or broad intellectual function but differed in years of education, employment, and household income (**Table 2**); partial responders also had a longer history of methamphetamine use and demonstrated more problematic use of alcohol (at trend levels) than full responders. The inclusion of covariates did not explain significantly more variance than a model in absence of covariates for both the magnitude effect (likelihood ratio = 4.94, *p* = 0.293) and frequency effect (likelihood ratio = 5.39, *p* = 0.249), and the inclusion of covariates did not improve the precision of estimates. In turn, covariates were excluded, and simpler models were retained. Additional demographics of interest are included in **Table 2**.

**Table 2:** Full sample characteristics (N = 49).

Variable	Partial responders (n = 12)	Full Responders (n = 17)	Healthy Controls (n = 20)	p
Sociodemographic characteristics				
Age, mean (SD)	34.83 (5.62)	33.76 (6.68)	34.95 (6.36)	0.835
Gender (M: F)	10:2	10:7	13:7	0.370
Education	9.58 (2.42)	11.11 (2.9)	12.25 (1.05)	0.001*
Employment (Y: N)	0:12	^a^4:12	12:8	0.002*
Household income (RAND), mean (SD)	40417 (27672)	14118 (19404)	22375 (21018)	0.011*
Cognitive characteristics				
WASI IQ, mean (SD)	87.33 (12.57)	91.47 (21.55)	86.35 (15.42)	0.653
Cigarette use				
Cigarettes smoked/day, mean (SD)	10.66 (9.33)	6.82 (6.02)	7.40 (6.67)	0.335
Nicotine dependence, mean (SD)	3.00 (1.63)	3.58 (2.69)	2.75 (2.22)	0.542
Methamphetamine (MA) use history				
Duration of MA use (years), mean (SD)	12.75 (3.54)	9.88 (4.48)	–	0.076^+^
Baseline MA negative, %	58.20 (22.10)	63.70 (19.60)		0.483
Previous MA stop attempts (n), mean (SD)	2.91 (3.14)	3.70 (5.93)	–	0.678
MA and substance use severity				
MA use quantity (grams), mean (SD)	1.14 (0.71)	0.87 (0.48)	–	0.235
Drug use severity, mean (SD)	0.25 (0.06)	0.26 (0.09)		0.995
Alcohol use severity, mean (SD)	0.10 (0.01)	0.08 (0.02)	–	0.074^+^
Other substance use and concurrent treatment				
Secondary substance (Methaqualone &/or cannabis: none)	9:3	^a^8:8	–	0.342
Concurrent outpatient treatment (Y: N)	9:3	9:8	–	0.413

MA = methamphetamine. Employment = Binary (yes or no) variable representing current employment. a = missing value/s in total sample. Household income = Yearly household income variable (R14: $1 US) derived from an ordinal 5 income category variable, where average income was extracted from the income range reflected within an income category. WASI IQ = aggregate score derived from both verbal and performance subsets of the Weschler-abbreviated scale of intelligence test. Nicotine dependence = measured using the Fagerström test. Baseline MA negative= proportion of MA-negative tests during baseline period prior to CM treatment. Previous MA stop attempts = Frequency of previous attempts to abstain from MA. Drug (and Alcohol) use severity = composite scores derived from the addiction severity index. Secondary substance = binary variable (Methaqualone &/or cannabis or none) indicating presence or absence of use of specific secondary substances besides MA. Concurrent outpatient treatment = binary variable (yes or no) indicating concurrent participation in motivational interviewing and/or group therapy alongside CM. F tests were utilized to assess potential group differences in Age, Education, Household income, WASI IQ, Cigarettes smoked/day, Nicotine dependence, Duration of MA use, Baseline MA negative, Previous MA stop attempts, MA use quantity as well as Drug and Alcohol use severity. Fisher’s exact tests were conducted on count factors including; gender and employment, whilst chi-squared tests were conducted on Concurrent outpatient treatment and Secondary substance. ^+^p < 0.10, *p < 0.05, **p < 0.01, ***p < 0.001

#### Magnitude Effect

**Figure 1:**
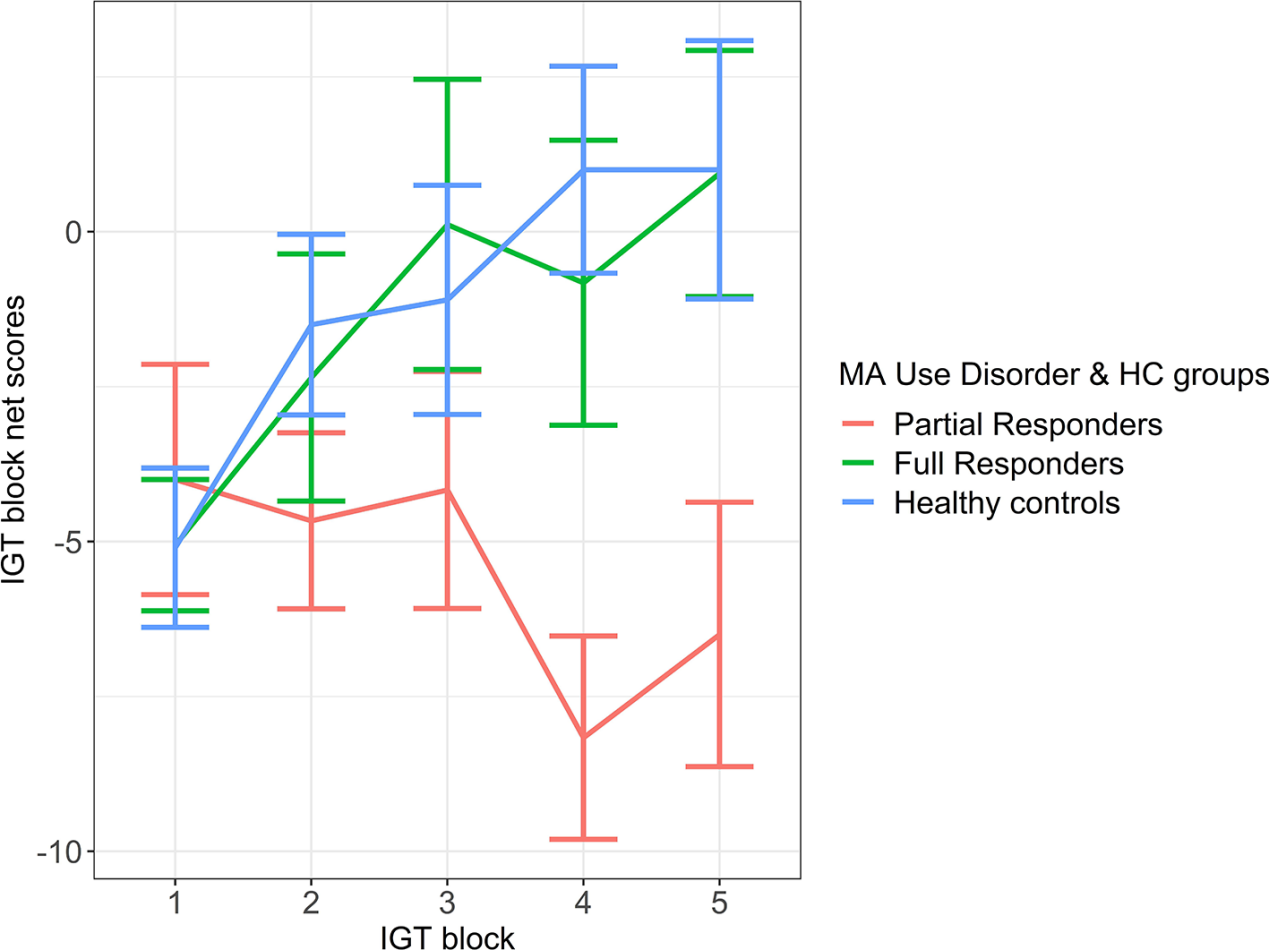
Magnitude Effect. IGT net scores by block for partial responder, full responder and control groups. HC, healthy control. Displays relative mean group differences in Magnitude Effect (where error bars represent standard error), measured by the preference for large, short term rewards over long term gains across the entire duration of the IGT. Lower block scores represent a higher Magnitude Effect, where riskier choices associated with large, immediate rewards are favoured whilst higher block scores represent a lower Magnitude Effect, illustrated by a greater tendency to avoid risky choices and select safer decks tied to lower, short term rewards but higher long-term gains.

An LME model of magnitude effect demonstrated significantly greater fit over a fixed-effect model (likelihood ratio = 49.62, *p* < 0.001), suggesting the impact that individual variability plays in estimating the magnitude effect. Group contrasts from the LME magnitude effect model demonstrated a significant difference between partial responders and healthy controls in magnitude effect, with a large effect size. More specifically, partial responders favored decks tied to large, short-term reward and withstood long-term loss more than healthy controls (**Figure 1** and **Table 3**). Partial responders also appeared to favor large, immediate rewards and withstood future losses more than full responders, although this finding was at trend level. Conversely, full responders did not differ from healthy controls in magnitude effect. Interestingly, while partial responders performed most poorly in magnitude effect on average [mean (SE) = -5.87 (1.79)], full responders and healthy controls did not score above chance level [mean (SE) = -0.52 (1.50) and -0.15 (1.38), respectively], with an average net gain around zero. See Appendix A of **Datasheet 1 (Supplementary Material)** for LME model estimates.

**Table 3:** Group contrasts from LME Magnitude Effect model on baseline IGT.

Contrasts	Mmd	g (CI)	p
Partial responders– Controls	-5.72	-0.77 (-1.09: -0.44)	0.038*
Full responders – Controls	-0.37	-0.04 (-0.38: 0.26)	0.981
Partial responders – Full responders	-5.34	-0.67 (-1.05: -0.35)	0.067^+^

mmd, marginal mean difference between groups. g = hedges g effect size. Tukey’s p-adjustment used to correct for multiple comparisons. +p < 0.10, *p < 0.05, **p < 0.01, ***p < 0.001.

#### Frequency Effect

**Figure 2:**
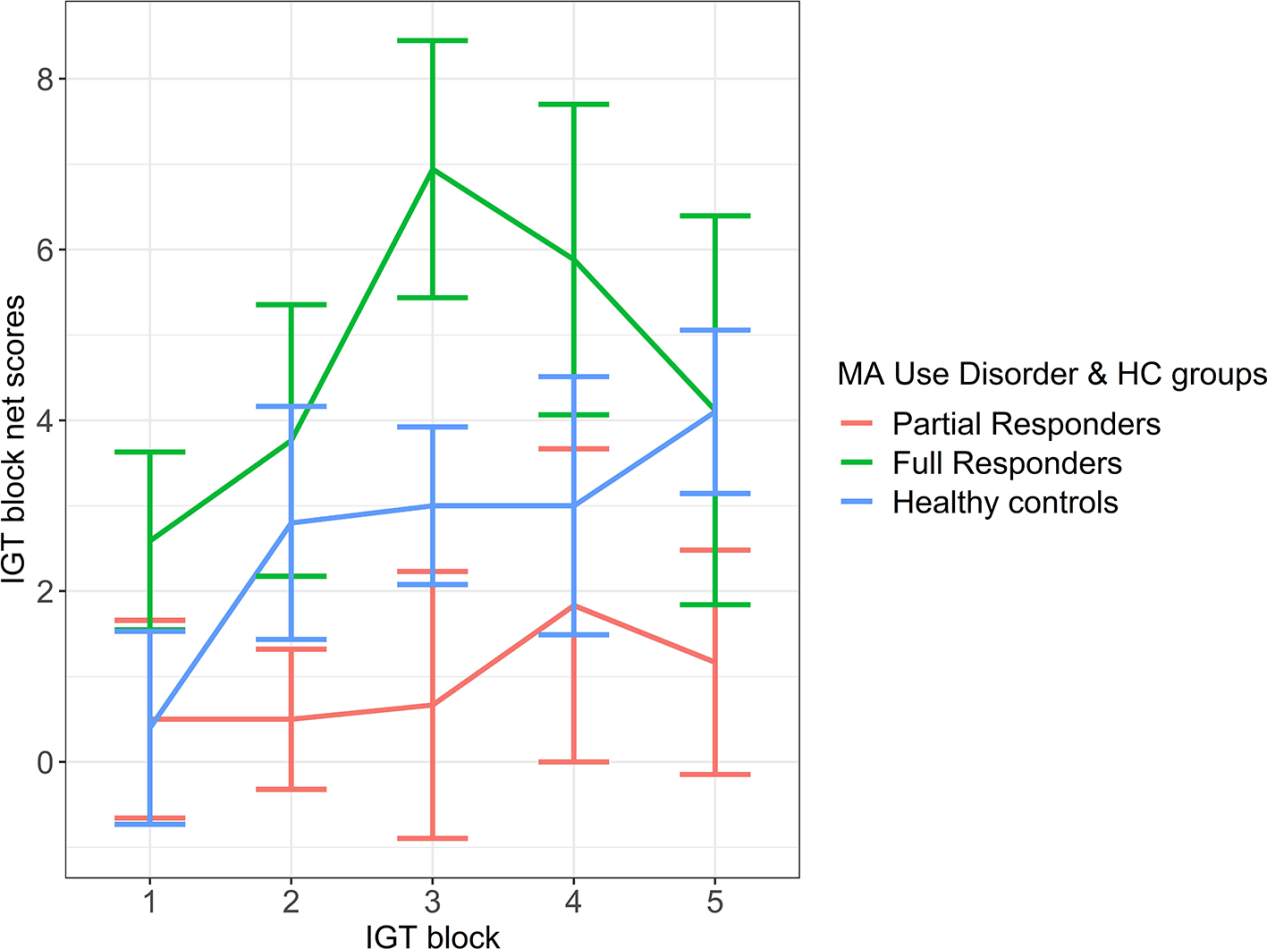
Frequency effect: IGT net scores by block for partial responder, full responder and control groups. HC, healthy control. Displays relative mean group differences in Frequency Effect (where error bars represent standard error), measured by the preference for frequent rewards and avoidance of frequent loses across the entire duration of the IGT. Higher block scores represent higher Frequency Effect, where frequent rewards are favoured and frequent losses are avoided, whilst lower block scores represent a lower Frequency Effect. Illustrated by a relatively diminished tendency to favour frequent rewards and avoid frequent losses.

An LME model of the frequency effect was significantly improved over a fixed-effect model (likelihood ratio = 38.35, *p* < 0.001). On the LME frequency effect model, a group difference in frequency effect was exhibited between full responders [mean (SE) = 5.18 (1.11)] and partial responders [mean (SE) = 1.04 (1.33)], where full responders demonstrated a greater tendency than partial responders to favor frequent rewards and avoid frequent losses (**Figure 2**). However, this difference was at trend level with a moderate effect size (**Table 4**). Healthy controls [mean (SE) = 3.23 (1.03)] did not differ from full responders or partial responders on the frequency effect. See Appendix B of **Datasheet 1 (Supplementary Material)** for LME model estimates.

**Table 4:** Group contrasts from LME Frequency Effect model on baseline IGT.

Contrasts	Mmd	g (CI)	p
Partial responders– Controls	-2.18	-0.42 (-0.74: -0.08)	0.401
Full responders – Controls	1.95	-0.30 (-0.01: 0.66)	0.409
Partial responders – Full responders	-4.13	-0.63 (-0.95: -0.29)	0.054^+^

mmd = marginal mean difference between groups. g = hedges g effect size. CI = confidence interval. Tukey’s p-adjustment used to correct for multiple comparisons. ^+^p < 0.10, *p < 0.05, **p < 0.01, ***p < 0.001.

## Discussion

Individuals with MA Use Disorder, who did not respond fully to CM treatment, demonstrated maladaptive decision-making that was characterized by a greater preference for risky choices associated with large, immediate rewards and long-term losses, over safer choices that were associated with greater long-term gain, relative to healthy controls. This finding points to the importance of the *immediacy* and *size* of reward outcomes, in conjunction with a lack of consideration for and/or limited ability to hold long-term consequences in mind, and their links to maladaptive decision-making and ultimately poor performance, otherwise referred to as the magnitude effect. Moreover, our findings are supported by literature findings of poor IGT performance in samples of individuals with drug use problems other than methamphetamine (1, 3, 4), as well as those who suffer from MA Use Disorder (2, 5).

Given that the magnitude effect incorporates the potential influence of the *magnitude* as well as the *immediacy* of the outcome on decision-making, impaired performance on the IGT could be driven by a prepotent drive for large, immediate gains in spite of both short-term and long-term negative consequences (1), or an insensitivity to future consequences despite the valence, as demonstrated in patients with ventromedial prefrontal cortical lesions (13). Using a modified IGT version, Bechara et al. (3) found that impaired performance by substance users is typically driven by a desire to seek rewards rather than an insensitivity to future consequences. This view is supported by findings that MA Use Disorder patients exhibit greater temporal discounting of reward value than healthy controls, illustrating a preference for immediate gains (8, 39). In turn, findings regarding temporal discounting suggest that maladaptive decision-making by partial responders is likely to be predominately driven by the tendency to favor large, immediate rewards in particular. Future studies with larger sample sizes should address the impact of *immediacy* and *magnitude* subcomponents of the IGT on decision-making.

Healthy controls and individuals who fully responded to CM performed at chance level (net zero gain) with respect to the magnitude effect, which is in contrast to previous studies of IGT performance by healthy individuals in particular, where healthy individuals were found to be able to obtain net positive gains on the IGT (1, 3). This contrast in findings may be explained by differing sample characteristics, where previous studies predominately consisted of university-educated healthy individuals, while the current study consisted of education-matched healthy controls with a lower, secondary-level education on average.

Decision-making among healthy samples is also influenced by the frequency with which rewards and losses are presented (10, 11). In this study, healthy controls did not differ from full responders or partial responders in the frequency effect, but full responders appeared to favor frequent rewards and avoided frequent losses more often than partial responders. This observation suggests that full responders and partial responders may present with differing decision-making profiles. In the context of the IGT where optimal decision-making is represented by a tendency to favor long-term gains in spite of small, immediate rewards, a tendency to favor frequent rewards and avoid frequent losses represents a kind of suboptimal decision-making strategy. Favoring of the frequency effect might be somewhat maladaptive in the context of the IGT, although full responders’ task-based preference to receive frequent rewards and to avoid frequent losses in this pilot study may correspond with a responsiveness toward frequent positive reinforcement with monetary vouchers from sustained abstinence from methamphetamine.

Baseline IGT performance differences associated with response to CM indicate that an individual’s cognitive strategy for balancing reward and potential loss can be an important factor to consider in deciding whether CM is the best treatment for a particular client. The very nature of CM, which involves forgoing immediate gain (from drug use) for a greater long-term gain (vouchers for abstinence), is consistent with greater therapeutic success of clients who can avoid immediate, large rewards that carry the risk of long-term loss. The findings also point to the influence of the frequency with which such decision alternatives arise. Future work confirming links between maladaptive decision-making and outcomes of CM treatment for MA Use Disorder might offer quick, affordable methods to separate persons most likely to fully respond from those who respond relatively less so to CM.

There are several limitations in this study. Sample size was small, but hypothesized meaningful findings were still obtained, and so was sufficient in size to test hypotheses. Groups were not perfectly matched against all potentially relevant sociodemographic, cognitive, and drug-use factors that may covary with performance, and models were run in absence of any covariates, which could lead to under- or overestimation of model estimates in small samples. Steps were taken to increase the precision of model estimates with use of LME models, which account for potential confounding effects of individual differences in performance. Moreover, groups were not examined on executive functioning capabilities, which have been strongly tied to performance on IGT (2, 5). As such, group differences in performance may partly be explained by executive functioning differences. However, a review by Toplak et al. (40) found that performance on the IGT was weakly related to various cognitive capabilities. A flat rate monetary incentive was used for task performance, instead of a performance-sensitive monetary incentive, due to logistical limitations of obtaining customized monetary vouchers. However, this flat rate was consistently applied across partial responder, full responder, and controls groups. Lastly, IGT findings cannot necessarily be uniquely tied to CM treatment, and future studies should compare the relationship between IGT performance and CM to that of other treatment types.

## Conclusion

Partial responders to CM exhibited maladaptive decision-making as compared with healthy controls, reflected by the favoring of large, immediate rewards over long-term gains—the magnitude effect. Partial responders and full responders also appeared to differ in frequency effect, where full responders demonstrated a greater preference for frequent rewards and avoided frequent losses more than partial responders. Evidence of group differences in magnitude effect and frequency effect suggests a difference in decision-making profiles, with different associated implications for treatment response on CM. In particular, the finding that the magnitude effect was more linked to lowered response to CM whereas the frequency effect was associated with positive response suggests that the magnitude effect is a risk factor for relapse during CM treatment, whereas the frequency effect may act as a cognitive strategy that predicts greater CM treatment success in the form of sustained abstinence.

## Data Availability Statement

The datasets generated for this study are available on request to the corresponding author.

## Ethics Statement

The studies involving human participants were reviewed and approved by Health Sciences Human Research Ethics committee of the University of Cape Town and UCLA Institutional Review Board. The patients/participants provided their written informed consent to participate in this study.

## Author Contributions

ML conceived the study focus with support from JI. SS conceived the broader study design with additional contributions from LN, EL, and DS. LN project managed the broader study and was in charge of data acquisition with assistance from ML. ML conducted analysis of data. ML interpreted findings of data with assistance from JI. ML wrote up the paper; revisions were obtained from all authors, with the biggest contributions from EL, ST, and SS. Final approval of the manuscript was obtained from all authors.

## Funding

This work was supported by the National Institute on Drug Abuse R21DA040492-01 [FAIN No. R21DA040492] and the Department of Psychiatry and Mental Health, University of Cape Town. ST also acknowledges salary support by the VA Office of Academic Affiliations through the National Clinician Scholars Program. SS acknowledges salary support by the National Institute of Mental Health P30 058107—Center for HIV Identification, Prevention and Treatment Services, University of California, Los Angeles, Center for AIDS Research grant AI028697. DJS acknowledges salary support by the South African Medical Research Council.

## Disclaimer

The contents do not represent the views of the South African government, U.S. Department of Veterans Affairs or the United States Government.

## Conflict of Interest

The authors declare that the research was conducted in the absence of any commercial or financial relationships that could be construed as a potential conflict of interest.
