# Supplementary material for: Decision-Making by Patients With Methamphetamine Use Disorder Receiving Contingency Management Treatment: Magnitude and Frequency Effects
Source: Front Psychiatry. 2020 Feb 18;11:22. doi: 10.3389/fpsyt.2020.00022 (PMC7058183; doi:10.3389/fpsyt.2020.00022)
Supplement: Supplementary file 1 [file DataSheet_1.docx]

**Appendix A**

| **Table S1** | | |  |  |
| --- | --- | --- | --- | --- |
| Magnitude effect on IGT at baseline: LME model estimates | | |  |  |
| Parameter | Estimate | CI | | *p* |
|  |  |  | |  |
| Fixed effects |  |  | |  |
| Intercept (Partial responders) β_0_ | -5.87 | (-9.40: -2.34) | | 0.001* |
| Full responders β_1_  Healthy controls β_2_ | 5.34  5.72 | (0.64: 10.04)  (1.17: 10.27) | | 0.026^+^  0.014* |
|  |  |  | |  |
| Random effects |  |  | |  |
| Intercept variance α_0_ | 5.48 | (4.21: 7.12) | |  |
| Error variance σ^2^_ɛ_ | 5.76 | (5.14: 6.46) | |  |
| *Note.* CI = 95% confidence interval. ^+^ *p<.*10, * *p* < .05, ** *p* <.01, *** *p* <.001. Model fit: Likelihood ratio chi-square= 7.23, p=0.026. Omega-squared = 0.59 | | | | |

**Appendix B**

| **Table S2** | | |  |  |
| --- | --- | --- | --- | --- |
| Frequency effect on IGT at baseline: LME model estimates | | |  |  |
| Parameter | Estimate | CI | | *p* |
| Fixed effects |  |  | |  |
| Intercept (Partial responders) β_0_ | 1.04 | (-1.58: 3.66) | | 0.433 |
| Full responders β_1_  Healthy controls β_2_ | 4.13  2.18 | (0.64: 7.62)  (-1.19: 5.56) | | 0.021*  0.199 |
|  |  |  | |  |
| Random effects |  |  | |  |
| Intercept variance | 3.95 |  | |  |
| Error variance | 4.67 |  | |  |
| *Note.* CI = 95% confidence interval. ^+^ *p<.*10, * *p* < .05, ** *p* <.01, *** *p* <.001.  Model fit: Likelihood ratio chi-square= 5.75, p=0.056. Omega-squared = 0.53 | | | | |
